# Evaluation of BOX-PCR and ERIC-PCR as Molecular Typing Tools for Pathogenic *Leptospira*

**DOI:** 10.1155/2018/1351634

**Published:** 2018-08-01

**Authors:** Lesley Maurice Bilung, Chai Fung Pui, Lela Su'ut, Kasing Apun

**Affiliations:** ^1^Faculty of Resource Science and Technology, Universiti Malaysia Sarawak, 94300 Kota Samarahan, Sarawak, Malaysia; ^2^Faculty of Medicine and Health Sciences, Universiti Malaysia Sarawak, 94300 Kota Samarahan, Sarawak, Malaysia

## Abstract

In the last decades, leptospirosis had gained public health concern due to morbidity and mortality rates caused by pathogenic *Leptospira*. The need for rapid and robust molecular typing methods to differentiate this zoonotic pathogen is of utmost importance. Various studies had been conducted to determine the genetic relatedness of *Leptospira* isolates using molecular typing methods. In this study, 29 pathogenic *Leptospira* isolates from rat, soil, and water samples in Sarawak, Malaysia, were characterized using BOX-PCR and ERIC-PCR. The effectiveness of these two methods with regard to the ease of interpretation, reproducibility, typeability, and discriminatory power was also being evaluated. Using BOX-PCR, six clusters and 3 single isolates were defined at a genetic distance percentage of 11.2%. ERIC-PCR clustered the isolates into 6 clusters and 2 single isolates at a genetic distance percentage of 6.8%. Both BOX-PCR and ERIC-PCR produced comparable results though the discriminatory index for ERIC-PCR (0.826) was higher than that for BOX-PCR (0.809). From the constructed dendrogram, it could be summarized that the isolates in this study were highly heterogeneous and genetically diverse. The findings from this study indicated that there is no genetic relatedness among the pathogenic *Leptospira* isolates in relation to the locality, source, and identity, with some exceptions. Out of the 29 pathogenic *Leptospira* isolates studied, BOX-PCR and ERIC-PCR successfully discriminated 4 isolates (2 isolates each) into the same cluster in relation to sample sources, as well as 2 isolates into the same cluster in association with the sample locality. Future studies shall incorporate the use of other molecular typing methods to make a more thorough comparison on the genetic relatedness of pathogenic *Leptospira*.

## 1. Introduction

Leptospirosis is a worldwide zoonotic disease caused by pathogenic *Leptospira*. Traditionally, the genus *Leptospira* is divided into two species, namely, *L. interrogans* comprising pathogenic strains and *Leptospira biflexa* comprising saprophytic strains [1]. Currently, DNA-DNA hybridization studies classified *Leptospira* into 21 species. Phylogenetic analysis of *Leptospira* further clustered them into three clades designated as pathogenic, intermediate, and nonpathogenic [2, 3]. Based on the lipopolysaccharide antigenic classification, more than 250 serovars that belong to at least 24 serogroups have been determined [4].

Rapid identification and characterization of isolated *Leptospira* can contribute to the surveillance of local serovars, tracking for a novel pattern of disease presentation and development of intervention measures [5]. Serological typing using cross agglutinin absorption test (CAAT) is the reference method for serovar identification. However, this method is laborious due to the requirement for extensive collection of reference antisera and live antigens [6]. Therefore, microscopic agglutination test (MAT) is the serological method used to identify *Leptospira* isolates at the serogroup level [7]. Nowadays, molecular typing methods are used as alternative typing methods. According to Fouts and coworkers [8], multilocus sequencing typing (MLST) and pulsed field gel electrophoresis (PFGE) are the two commonly used molecular typing methods for *Leptospira*.

Repetitive element-based PCR (Rep-PCR) has been widely used to study the strain-specific patterns obtained from PCR amplification of repetitive DNA elements present within bacterial genomes [9, 10]. The advantages of Rep-PCR over other molecular typing methods include the ability to differentiate between closely related strains of bacteria, as well as being a simple, quick, inexpensive, and reliable high-throughput genotyping method [11, 12].

Two of the repetitive elements used for molecular typing are BOX elements and enterobacterial repetitive intergenic consensus (ERIC) sequences [13, 14]. BOX elements are mosaic repetitive elements comprised of different combinations of three subunit sequences. These three subunit sequences are boxA, boxB, and boxC which are 59, 45, and 50 nucleotides long, respectively [15, 16]. Meanwhile, ERIC sequences are 126 bp long with a highly conserved central inverted repeat. It is situated in noncoding transcribed regions of the chromosome [17]. Briefly, the BOX primer anneals on the boxA subunit of BOX elements whereas the ERIC primer synthesises DNA sequences outward from inverted repeats [18].

In this study, we attempted to determine the genetic relatedness of pathogenic *Leptospira* isolated from rats and environments in Sarawak, Malaysia. This is the first publication evaluating the effectiveness and robustness of BOX-PCR and ERIC-PCR as molecular typing tools for pathogenic *Leptospira*.

## 2. Materials and Methods

### 2.1. Bacterial Strains

A total of 29 pathogenic *Leptospira* species, isolated from rats, soil, and water samples from different localities in Sarawak, were examined in this study (Table 1). Sampling permit (permit number NCCD.907.4.4 (Jld.10)-185) was obtained from Sarawak Forestry Corporation for the Forest Park Entrance Permit to collect samples from national parks and wildlife sanctuary. The Commission of the City of Kuching North approved the sampling in urban areas of Sarawak (reference number DBKU/ENV/CSA/2/25(35)). Sample collection from national service training centres was permitted by the National Service Training Department and the camp managers of selected national service training centres (reference number KP/JLKN(TADBIR BUKP)61 JIL.9(20)). *L. noguchii* and *L. interrogans* were used as positive controls. All the strains were cultured at 30°C in modified semisolid Ellinghausen-McCullough-Johnson-Harris (EMJH) media with 100 *μ*g/mL 5-fluorouracil.

### 2.2. Genomic DNA Extraction

Genomic DNA was extracted using Wizard™ Genomic DNA Purification Kit (Promega Corporation, USA) following the manufacturer's instructions.

### 2.3. BOX-PCR

The primer BOXA1R (5′-CTACGGCAAGGCGACGCTGACG-3′) was used for BOX-PCR fingerprinting. The 25 *μ*L reaction mixture contained 5 *μ*L of 5x PCR buffer, 400 *μ*M of deoxynucleoside triphosphate mix, 0.4 *μ*M of primer, 3 mM of magnesium chloride, 2.5 U of *Taq* DNA polymerase (Promega Corporation, USA), and 5 *μ*L of DNA template. PCR condition included initial denaturation at 94°C for 5 min, followed by 35 cycles of denaturation at 94°C for 1 min, primer annealing at 40°C for 2 min, and extension at 72°C for 2 min, with a final extension at 72°C for 10 min.

### 2.4. ERIC-PCR

The 29 isolates were fingerprinted by ERIC-PCR using the primer set ERIC 1R (5′-ATGTAAGCTCCTGGGGATTCA C-3′) and ERIC 2 (5′-AAGTAAGTGACTGGGGTGAGCG-3′). 5 *μ*L of DNA template was added to 25 *μ*L reaction mixture with 5 *μ*L of 5x PCR buffer, 200 *μ*M of deoxynucleoside triphosphate mix, 1 *μ*M of each primer, 3 mM of magnesium chloride, and 1 U of *Taq* DNA polymerase (Promega Corporation, USA). PCR conditions used were initial denaturation at 95°C for 5 min, followed by 35 cycles of denaturation at 94°C for 30 sec, primer annealing at 47°C for 30 sec and 52°C for 1 min, and extension at 72°C for 4 min, with a final extension at 72°C for 16 min.

### 2.5. Agarose Gel Electrophoresis

The PCR products were fractionated by electrophoresis using 2% agarose gel in a 1x TBE buffer. The gel was stained with ethidium bromide and viewed under an ultraviolet (UV) transilluminator. A 1 kb DNA ladder (Thermo Fisher Scientific, USA) was included in each gel as a molecular weight marker.

### 2.6. Cluster Analysis

The banding patterns generated by BOX-PCR and ERIC-PCR were analysed using PyElph version 1.4. The dendrograms were constructed using an unweighted pair group method with arithmetic mean (UPGMA), according to published guidelines by Pavel and Vasile [19]. We chose this method because UPGMA is the simplest distance-matrix method in constructing a phylogenetic tree using uncorrected data.

### 2.7. Discriminatory Index (*D*)

The discriminatory indices (*D*) of BOX-PCR and ERIC-PCR at selected genetic distance percentages were calculated based on Simpson's Index of Diversity using the formula described by Hunter and Gaston [20]:

(1) $$\begin{array}{c} D=1-\frac{1}{N\left(N-1\right)}\overset{S}{\underset{j=1}{\sum }}n_{j}\left(n_{j}-1\right), \end{array}$$

where *N* is the total number of strains in the sample population, *S* is the total number of types described, and *n*_*j*_ is the number of strains belonging to the *j*th type. A value of 1 is highly discriminatory, and a value of 0 is not discriminatory.

## 3. Results

**Figure 1:**
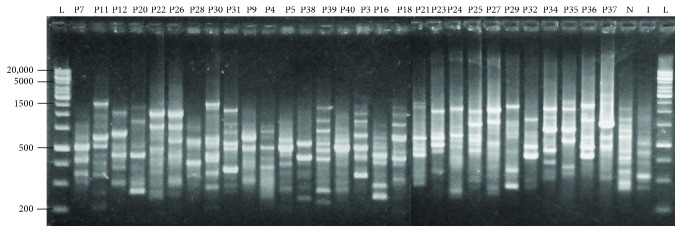
BOX-PCR profile of 29 pathogenic *Leptospira* isolates using PyElph software. The genetic distances are shown above the branches. Lane L denotes 1 kb DNA ladders, lane N denotes positive control (*L. noguchii*), and lane I denotes positive control (*L. interrogans*).

**Figure 2:**
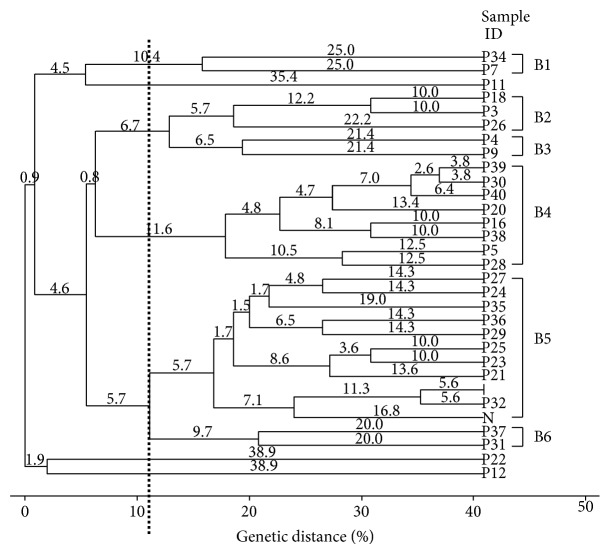
Dendrogram generated from BOX-PCR fingerprinting of 29 isolates of pathogenic *Leptospira* isolates. Six clusters and 3 single isolates were categorized at a genetic distance of 11.2% (boxed lines).

All the primers used in this study generated genomic fingerprinting patterns for all the isolates examined as depicted in Figures 1 and 2. The discriminatory indices of BOX-PCR and ERIC-PCR at different genetic distance percentages in genotyping of 29 pathogenic *Leptospira* isolates were summarized in Table 2.

Molecular typing of pathogenic *Leptospira* isolates using the BOXA1R primer generated 4 to 11 bands ranging from 200 to 20,000 bp (Figure 1). From Table 2, it was summarized that BOX-PCR clustered the isolates into 4, 6, and 6 clusters, with discriminatory indices 0.665, 0.809, and 0.862, at genetic distance percentages of 6.3%, 11.2%, and 16.9%, respectively. Major clusters were observed at a genetic distance percentage of 11.2% (Figure 2). The discriminatory index of 0.809 indicated that the overall similarity among the 29 isolates was only 19.1%. Six clusters were categorized at a genetic distance percentage of 11.2%. Most isolates were found in cluster B5, with 11 isolates (35.5%), and cluster B4, with 8 isolates (25.8%). This was followed by 3 isolates (9.7%) in cluster B2 and 2 isolates (6.5%) each in clusters B1, B3, and B6. Three isolates (P11, P12, and P22) were found to be single unique isolates.

**Figure 3:**
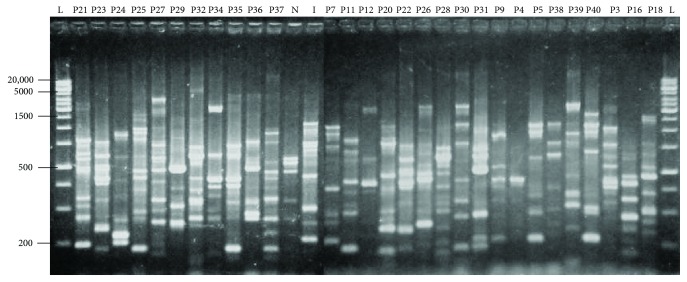
ERIC-PCR profile of 29 pathogenic *Leptospira* isolates using PyElph software. The genetic distances are shown above the branches. Lane L denotes 1 kb DNA ladders, lane N denotes positive control (*L. noguchii*), and lane I denotes positive control (*L. interrogans*).

**Figure 4:**
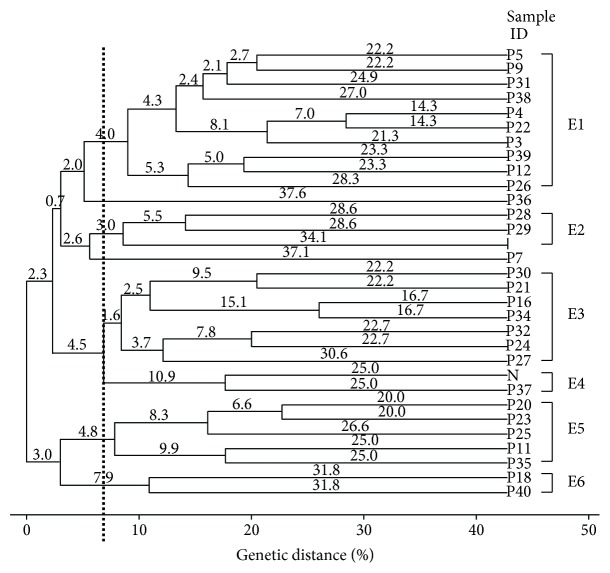
Dendrogram generated from ERIC-PCR fingerprinting of 29 isolates of pathogenic *Leptospira* isolates. Six clusters and 2 single isolates were categorized at a genetic distance of 6.8% (boxed lines).

ERIC-PCR of 29 pathogenic *Leptospira* isolates yielded different banding patterns to produce between 2 and 13 bands ranging from 200 to 20,000 bp (Figure 3). Table 2 indicates that ERIC-PCR clustered the isolates into 6, 9, and 10 clusters, with discriminatory indices 0.826, 0.856, and 0.897, at genetic distance percentages of 6.8%, 10.0%, and 13.3%, respectively. It was noticed that major clusters were defined at a genetic distance percentage of 6.8% (Figure 4). The overall similarity among the 29 isolates was 17.4% since the discriminatory index for ERIC-PCR was 0.826. Majority of the isolates were delineated into cluster E1, containing 10 isolates (32.3%). There were 7 isolates (22.6%) in cluster E3, 5 isolates (16.1%) in cluster E5, 3 isolates (9.7%) in cluster E2, and 2 isolates (6.5%) each in clusters E4 and E6. Two isolates (P7 and P36) were not grouped into any cluster.

## 4. Discussion

To the best of the authors' knowledge, no publication on the molecular typing of *Leptospira* using BOX-PCR and ERIC-PCR had been reported. Nevertheless, molecular typing of *Leptospira* using other fingerprinting methods had been documented. Random amplified polymorphic DNA (RAPD) PCR was used by Corney et al. [21], Ramadass et al. [22], and Benacer et al. [23]. MLST was applied by Ahmed et al. [24] for the same purpose. It is well known that molecular characterization using BOX-PCR and ERIC-PCR had been studied for other bacteria. Mishra et al. [16] concluded the suitability and reproducibility of BOX-PCR and ERIC-PCR for the genetic discrimination of *Fusarium oxysporum* isolates. Michelim et al. [25] also reported that BOX-PCR and ERIC-PCR were able to discriminate clinical isolates of *Proteus mirabilis*. Apart from that, Syrmis et al. [13] proved that BOX-PCR and ERIC-PCR are powerful surveillance tools to characterize clinical *Pseudomonas aeruginosa* isolates recovered from patients with cystic fibrosis.

The effectiveness of BOX-PCR in a genetic relatedness study of different bacteria had been evaluated by many researchers. For example, Lanoot et al. [26] revealed BOX-PCR as a powerful tool in fingerprinting 473 *Streptomyces* species. Results obtained by Marques et al. [27] supported the fact that BOX-PCR can discriminate 120 bacterial strains belonging to the *Pseudomonas syringae*-*P. viridiflava* group at a species level. On the other hand, various genotyping studies on different bacteria using ERIC-PCR had also been conducted. Tanil et al. [28] characterized *Vibrio parahaemolyticus* isolates using ERIC-PCR. The performance of ERIC-PCR in molecular typing of 116 *Shigella* isolates was evaluated by Kosek et al. [29] while Candan et al. [30] studied the genetic relatedness of *Staphylococcus aureus* strains from various clinical samples using ERIC-PCR.

Some criteria to consider in the evaluation of molecular typing methods are ease of interpretation, reproducibility, typeability, and discriminatory power [31]. Ease of interpretation was composed of the overall performance of the method used, as well as the interpretation of the resulting data [32]. Since no molecular typing study on *Leptospira* using BOX-PCR and ERIC-PCR had been published to make comparison, the authors tried to interpret the data to the best of their knowledge. However, in utilizing molecular typing methods to discriminate *Lactobacillus*isolates from the chicken gastrointestinal tract, Stephenson et al. [12] stated that ERIC-PCR profiles are easy to interpret and therefore can be adapted to high-throughput analysis of isolates.

The ability of a method to give the same result when replicate assays are performed on the same isolate is known as reproducibility [31]. Many previous studies [9, 16, 26, 33] determined the reproducibility of the methods used by obtaining the same pattern from representative isolates several times. In our study, positive controls were incorporated each time we conducted BOX-PCR and ERIC-PCR fingerprinting. As the same fingerprinting pattern was obtained every time, it could be inferred that BOX-PCR and ERIC-PCR employed in this study were reproducible. A high level of reproducibility was also shown in genotyping of *Pseudomonas aeruginosa* isolates using BOX-PCR and ERIC-PCR [13].

Typeability is defined as the ability of a given method to provide a readable result for each isolate examined in a study [31]. Our result denoted that all the isolates produced bands after amplification by BOX-PCR and ERIC-PCR, which implied the complete typeability of pathogenic *Leptospira* isolates using these two molecular typing tools. This was corroborated by Dombek et al. [11] who described the complete typeability of *Escherichia coli* isolates using BOX-PCR in their study. Besides, Dorneles et al. [34] explained the high typeability of *Corynebacterium pseudotuberculosis* isolates using ERIC-PCR, where all the strains were being assigned a fingerprinting profile.

Discriminatory power is commonly determined by the discriminatory index (*D*). It is the quantitative measure of the probability of two unrelated strains being distinguished as different types [18]. In this study, the discriminatory indices for BOX-PCR and ERIC-PCR at different genetic distance percentages were determined. As the genetic distance percentages for BOX-PCR and ERIC-PCR increased, the discriminatory index also increased. As an example, for BOX-PCR, the discriminatory indices at genetic distance percentages 6.3% and 11.2% increased from 0.665 to 0.809.

According to Coenye et al. [32], higher discriminatory power does not always correspond to a more accurate representation of epidemiologic relatedness. This is because the effectiveness of a molecular typing method is not exclusively determined by the ability to discriminate the unrelated strains but also by the ability to form meaningful clustering [18]. As mentioned in Results, meaningful clustering for BOX-PCR was identified at a genetic distance percentage of 11.2% with a discriminatory index of 0.809. Meanwhile, at a genetic distance percentage of 6.8%, ERIC-PCR presented meaningful clustering with a discriminatory index of 0.826. As such, the higher discriminatory indices of 0.856 and 0.897 at genetic distance percentages of 10.0% and 13.3% were not chosen. Comparison between these two methods revealed the higher discrimination index for ERIC-PCR than BOX-PCR, but both of them gave comparable results.

Based on the BOX-PCR dendrogram constructed, it was found that the 29 isolates were genetically diverse and heterogeneous. Hence, there was no clear association between the identity of pathogenic *Leptospira* and fingerprinting profile. As the dominant species in this study, *L. noguchii* isolates were randomly resolved into clusters B2 (P18, P26), B4 (P16, P20, P38, P39, and P40), B5 (P21, P23, P25, P27, P32, P36, and positive control *noguchii*), B6 (P37), and single isolate P22. At a genetic distance percentage of 22.7%, it was noticed that P20 (water) and P38 (rat) shared identical BOX-PCR profiles in cluster B4. It was inferred that this closely related *Leptospira* species might be present in the rat and water sources in the same locality (national park) examined in this study. Besides, rats from urban areas (P39, P40) were distributed into the same cluster, which appeared to be closely related with genetic distance of 6.4. This reckoned the association between the sample source and locality in the two *L. noguchii* isolates.

Adding to this, *L. interrogans* isolates were also genetically heterogeneous as they were clearly distributed into clusters B3 (P4, P9, and P11), B4 (P5, P28, and P30), B6 (P31), and single isolate (positive control *interrogans*). With a genetic distance of 12.5, P5 (rat) and P28 (water) in cluster B4 were genetically closely related than P4 (rat) and P9 (water) with genetic distance of 21.4 in cluster B3, though they were all isolated from the same locality (urban areas). Nonetheless, the rats carrying the same *Leptospira* species in urban areas might have urinated and contaminated the water sources in the same locality. *L. borgpetersenii* isolates were heterogeneously observed at clusters B1 (P7, P34), B2 (P3), and B5 (P35). Two isolates from urban areas, P7 (water) and P34 (soil), formed a distinct cluster in cluster B1 with no other isolate being observed here. One soil sample from an urban site (P24) was discriminated into cluster B5 whereas one water sample from a national service training centre (P12) appeared as a single isolate, though both of them were *L. weilii*. Lastly, P29, being the only *L. santorosai* isolate, positioned itself in cluster B5, sharing a similarity with other *Leptospira* species.

Based on the ERIC-PCR dendrogram generated, there is also a lack of relatedness between the identity of pathogenic *Leptospira* and fingerprinting profile. This is because not all the 29 isolates were grouped into a specific cluster by species. *L. noguchii* isolates were distinguished into clusters E1 (P22, P26, P38, and P39), E3 (P16, P21, P27, and P32), E4 (P37, positive control *noguchii*), E5 (P20, P23, and P25), E6 (P18, P40), and single isolate P36. Among the 15 *L. noguchii* isolates, two of them (P22 and P26) were grouped into cluster E1 at a genetic distance percentage of 9.0% in relation to sample sources (water) and locality (paddy field). This suggested the possibility of closely related *Leptospira* strains being present in the water sources in the paddy field. Besides, all the *L. noguchii* isolates in cluster E3 were isolated from soil samples, unlike other clusters with heterogeneous sample sources.

Seven *L. interrogans* isolates were heterogeneously distributed into clusters E1 (P4, P5, P9, and P31), E2 (P28, positive control *interrogans*), E3 (P30), and E5 (P11). It was noticed that they differentiated themselves into different clusters according to localities. P30 from a national park in cluster E3 and P11 from a national service training centre in cluster E5 were not closely related to isolates from urban areas (P4, P5, P9, P28, and P31). All *L. interrogans* isolates from urban areas were distinguished into clusters E1 and E2 which belonged to the same cluster at a genetic distance percentage of 3.0%. It was also interesting to highlight that P4, P9, and P28 were water samples whereas P4 and P5 were rat samples. Since they were found to be closely related, it was hypothesized that leptospirosis could be transmitted through either direct contact with rat urine or indirect contact with water samples contaminated by rat urine at urban areas. There was no genetic similarity exhibited among the four *L. borgpetersenii* isolates (P3, P7, P34, and P35), two *L. weilii* isolates (P12, P24), and one *L. santarosai* isolate (P29).

Comparison between BOX-PCR and ERIC-PCR was done to find the similarity in clustering between these two molecular typing tools. It was observed that a few isolates were grouped into the same cluster for BOX-PCR and ERIC-PCR. The soil samples (P23 and P25) exhibited the same profile in cluster B5 of BOX-PCR and E5 of ERIC-PCR, which were both identified as *L. noguchii* isolates. Similarly, P21 and P32 both were isolated from soil samples, were indistinguishable, and could be observed at cluster B5 of BOX-PCR and E3 of ERIC-PCR, respectively. Consequently, it could be deduced that out of the 29 isolates in this study, 4 of them generated the same profile in both BOX-PCR and ERIC-PCR in association of sample sources. On the other hand, two isolates from urban areas, designated as P4 and P9, were grouped together at cluster B3 of BOX-PCR and E1 of ERIC-PCR. It is noteworthy that these two samples were highly related in relation to sample locality.

In this study, both BOX-PCR and ERIC-PCR gave comparable efficiency in determining the genetic relatedness of 29 pathogenic *Leptospira* isolates though a higher discriminatory index was obtained using ERIC-PCR (0.826) than BOX-PCR (0.809). Overall, these isolates could not be clearly discriminated into individual clusters based on different localities and sample sources. Although these two methods are easy to conduct in a short time, the findings in this study revealed the limited resolving power of BOX-PCR and ERIC-PCR in the determination of genetic relatedness among pathogenic *Leptospira*. It was inferred that no single molecular tool is ideal in typing different *Leptospira* species. However, combination of different molecular tools will differentiate them better and provide more thorough genetic relatedness data.

## 5. Conclusions

Strain discrimination of *Leptospira* isolates gives us a better understanding of the epidemiology of leptospirosis in a geographic region. A more thorough genetic relatedness data could be obtained by characterizing more isolates from the same localities and sources. In the future, different molecular typing tools such as RAPD, PFGE, and MLST can be compared to study the efficiency and effectiveness of these molecular typing tools in characterizing local *Leptospira* isolates.

## Figures and Tables

**Table 1:** Sample ID, source, and locality for 29 pathogenic *Leptospira* isolates examined in this study. UA: urban area.

Number	Sample ID	Species	Source	Locality
1	P3	*Leptospira borgpetersenii*	Soil	Bako National Park
2	P4	*Leptospira interrogans*	Rat liver	Universiti Malaysia Sarawak (UA)
3	P5	*Leptospira interrogans*	Rat kidney	Universiti Malaysia Sarawak (UA)
4	P7	*Leptospira borgpetersenii*	Water	Universiti Malaysia Sarawak (UA)
5	P9	*Leptospira interrogans*	Water	Universiti Malaysia Sarawak (UA)
6	P11	*Leptospira interrogans*	Water	Miri National Service Training Centre
7	P12	*Leptospira weilii*	Water	Samunsam Wild Life Sanctuary
8	P16	*Leptospira noguchii*	Soil	Miri National Service Training Centre
9	P18	*Leptospira noguchii*	Soil	Kubah National Park
10	P20	*Leptospira noguchii*	Water	Samunsam Wild Life Sanctuary
11	P21	*Leptospira noguchii*	Soil	Samunsam Wild Life Sanctuary
12	P22	*Leptospira noguchii*	Water	Sungai Mata Village (UA)
13	P23	*Leptospira noguchii*	Soil	Sungai Mata Village (UA)
14	P24	*Leptospira weilii*	Soil	Desa Ilmu (UA)
15	P25	*Leptospira noguchii*	Soil	Desa Ilmu (UA)
16	P26	*Leptospira noguchii*	Water	Plaie Village (UA)
17	P27	*Leptospira noguchii*	Soil	Plaie Village (UA)
18	P28	*Leptospira interrogans*	Water	Sebayor Village (UA)
19	P29	*Leptospira santarosai*	Soil	Sebayor Village (UA)
20	P30	*Leptospira interrogans*	Water	Gunung Gading National Park
21	P31	*Leptospira interrogans*	Water	Matang Village (UA)
22	P32	*Leptospira noguchii*	Soil	Medan Niaga Satok (UA)
23	P34	*Leptospira borgpetersenii*	Soil	Gita Village (UA)
24	P35	*Leptospira borgpetersenii*	Soil	Tupong Village (UA)
25	P36	*Leptospira noguchii*	Soil	Hui Sing (UA)
26	P37	*Leptospira noguchii*	Soil	Paya Mebi Village (UA)
27	P38	*Leptospira noguchii*	Rat liver	Gunung Gading National Park
28	P39	*Leptospira noguchii*	Rat kidney	Tupong Village (UA)
29	P40	*Leptospira noguchii*	Rat liver	Paya Mebi Village (UA)

**Table 2:** Discriminatory indices of BOX-PCR and ERIC-PCR in genotyping of pathogenic *Leptospira* isolates (*n* = 29).

Genotyping method	Genetic distance (%)	Number of clusters	Cluster sizes	Number of single isolate	Discriminatory index
BOX-PCR	6.3	4	2, 5, 8, 13	3	0.665
	11.2	6	2, 3, 2, 8, 11, 2	3	0.809
	16.9	6	3, 2, 8, 8, 3, 2	5	0.862

ERIC-PCR	6.8	6	10, 3, 7, 2, 5, 2	2	0.826
	10.0	9	7, 3, 2, 4, 3, 2, 3, 2, 2	3	0.856
	13.3	10	4, 3, 3, 2, 2, 2, 2, 2, 3, 2	6	0.897

## Data Availability

Data generated in this study are included in this article. The raw data generated using statistical analysis is available from the corresponding author on reasonable request.

## Abbreviations

CAAT: Cross agglutinin absorption test
*D*: Discriminatory indices
EMJH: Ellinghausen-McCullough-Johnson-Harris
ERIC: Enterobacterial repetitive intergenic consensus
MAT: Microscopic agglutination test
MLST: Multilocus sequencing typing
PFGE: Pulsed field gel electrophoresis
RAPD: Random amplified polymorphic DNA
Rep-PCR: Repetitive element-based PCR
UPGMA: Unweighted pair group method with arithmetic mean
UV: Ultraviolet.
